# A Possible Association between Melanoma and Prostate Cancer. Results from a Case-Control-Study

**DOI:** 10.3390/cancers7020670

**Published:** 2015-04-15

**Authors:** Alina Goldenberg, Shang I. Brian Jiang, Philip R. Cohen

**Affiliations:** 1 School of Medicine, University of California, San Diego, CA 92093, USA; E-Mail: alina8829@gmail.com; 2 Mohs Micrographic and Dermatologic Surgery, University of California, San Diego, CA 92122, USA; E-Mail: s2jiang@ucsd.edu; 3 Department of Dermatology, University of California, San Diego, CA 92122, USA

**Keywords:** melanoma, men, prevention, prostate cancer, screening

## Abstract

Melanoma and prostate cancer are the fifth and first most common cancers in men within the United States, respectively. The association between the two cancers lies in the mutual androgen-dependence. However, the relationship between prostate cancer history and melanoma development remains to be further elucidated. We aim to determine the odds of history of prostate cancer among men with melanoma as compared to time-frame, clinic, and provider-matched controls without melanoma within a single academic surgical center. We present a case-control study comparing men treated for melanoma and non-melanoma cancer by a single provider between 2010 and 2014 within an academic dermatologic surgical center. Overall, there were nine cases of prostate cancer among the melanoma group and two cases amongst the controls—a statistically significant difference in both uni- and multivariable analyses (*p* = 0.057 [95% CI 1, 23.5], *p* = 0.042 [95% CI 1.1, 129], respectively). Body mass index, alcohol use, and skin type II were significant risk factors for melanoma (*p* = 0.011 [95% CI 1, 1.3], 0.005 [95% CI 1.4, 7], 0.025 [95% CI 1.1, 3.3], respectively). There were more immunosuppressed controls (*p* = 0.002); however, the melanoma patients had a significantly longer duration of immunosuppression (11.6 *vs.* 1.9 years, *p* < 0.001 [95% CI 0.03, 0.5]). Melanoma screenings for men should include questions on prostate cancer history. Prostate cancer patients may benefit from more frequent and comprehensive melanoma screening.

## 1. Introduction

Melanoma is the fifth most common diagnosed cancer in men from the United States [1,2]. Melanoma incidence has been steadily rising over the past 30 years and many studies have focused on its risk factors in order to help promote and develop new treatments and prevention guidelines [2]. Melanoma pathogenesis is complex and involves both environmental and genetic factors. Intermittent ultraviolet exposure specifically periodic, intense sunlight (rather than long, continuous chronic exposure), is a major cause of melanoma. History of childhood blistering sunburns, used as a surrogate measurement for intermittent sun exposure, is a significant independent risk factor for melanoma development [3]. Light skin pigmentation, denoted by the Fitzpatrick skin types I–II, significantly increases the tendency to sunburn and thus, is also associated with increased melanoma risks [4,5].

Prostate cancer is the most common malignancy in men from the United States, it is also the second leading cause of death [2]. The association of an increased risk for melanoma among patients with prostate cancer has been reported in cancer registry data in 2011 and 2012 [6,7]. Recently, Li *et al.* confirmed these epidemiologic findings with a prospective cohort study with 42,372 participants [8]. The study data spanned over 20 years (1986 and 2010), and included 747,176 person-years. Within the time frame there were 5091 cases of prostate cancer and 539 new cases of melanoma. A personal history of prostate cancer was associated with an increased risk of melanoma in a multivariate-adjusted cox proportional hazards model with an adjusted hazard ratio of 1.83 (95% CI 1.32–2.54). The authors concluded that there was a significant association between melanoma and prostate cancer history possibly due to an underlying pathophysiologic overlap associated with androgens [8].

Nevertheless, the relationship between prostate cancer history and melanoma development remains to be further elucidated. The cohort used by Li *et al.* only included white health professionals, greatly limiting their generalizability to a greater ethnically diverse population, and allowing for possible socioeconomic confounders [8]. Thus, we propose a retrospective case-control of all male melanoma patients treated by a single provider within an academic institution over a 5-year period with the goals of assessing for any significant associations between prostate cancer and melanoma.

## 2. Results and Discussion

### 2.1. Results

**Figure 1:**
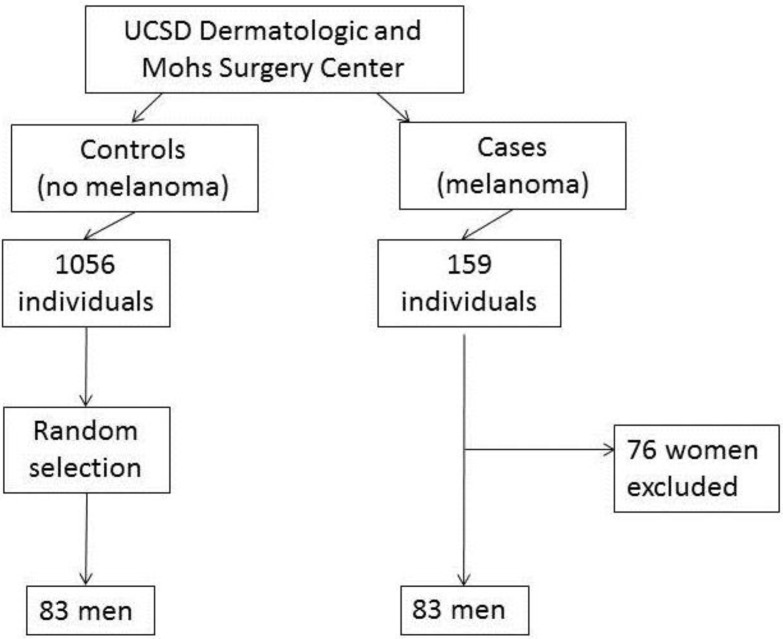
Study Design: 83 male cases of melanoma and 83 male cases of non-melanoma from a single provider within an academic center, treated within the same time period (03/2010–08/2014) were included in the study.

Over a four year period (03/2010–08/2014) 159 unique individuals were treated for melanoma within the University of California, San Diego Dermatologic and Mohs Surgery Center. Of these, 83 were men. Controls were chosen from 1056 individuals who underwent treatment within the same surgical center, by the same provider within an overlapping two year period between (03/2010–02/2012). Control and case groups each included 83 unique men equating to an overall cohort of 166 men (Figure 1).

The 83 melanomas within our cases represent 34 malignant melanomas, 45 melanomas *in-situ*, and 4 atypical junctional melanocytic proliferations; diagnoses were confirmed by a board-certified dermatopathologist. The majority of the melanomas were Clark level I (58.2%), with an overall mean Breslow depth of 0.36 mm, and range of 0.1 mm–0.60 mm. The majority of the cases were on the trunk (32.5%), and on the face (25.3%). In univariable analysis age, skin type, body mass index, cigarette use, pack years, alcohol use, benign prostatic hyperplasia medications, immunosuppression status, history of prostate cancer, and prostate cancer treatment were assessed for their potential to be risk factors for melanoma development (Table 1). Age was not significantly different between cases and controls with a mean of 67 years for melanoma males and 69 for non-melanoma males (*p* = 0.33). Fitzpatrick skin type II was the predominant skin type within both groups, however melanoma patients had 3.1 times the odds of having a skin type II than non-melanoma patients (*p* = 0.007 [95% CI 1.4, 7]). Additionally, skin type III patients had 0.34 times the odds of having melanoma than the controls (*p* = 0.047 [95% CI 0.07, 0.9]). Cases had significantly higher body mass index than the controls (*p* = 0.018), however both groups fell into the overweight category.

Current cigarette use, pack-years, Prostate-specific antigen, and benign prostatic hyperplasia medications were not significantly different between cases and controls. Melanoma cases were 3.2 times more likely to report alcohol use than controls (*p* < 0.001 [95% CI 1.7, 6.1]). Statistically significant larger number of controls were immunosuppressed than cases (29.1% *vs.* 6.4%, *p* < 0.001). Immunosuppressed patients with melanoma were immunosuppressed for a significantly longer duration than the immunosuppressed controls (*p* < 0.001). There were a total of 9 prior prostate cancers within the cases and 2 within the controls. Melanoma patients had 4.9 times the odds of having a prior history of prostate cancer than controls (*p* = 0.057 [95% CI 1, 23.5]).

Results were further adjusted in multivariable logistic regression for all of the significant risk factors found within the univariable analysis, and age (age, body mass index, skin type, alcohol use, immunosuppression, history of prostate cancer) (Table 2). Alcohol use and history of prostate cancer remained significant predictors of melanoma.

**Table 1:** Risk factors associated with melanoma development *.

Variable	Subvariable	Melanoma Cases N = 83	Non-Melanoma Controls ^a^ N = 83	OR	CI	*p*
Age (mean, SD)		67 (13)	69 (13.8)			0.332
Skin type N (%)	I	6 (7.2)	10 (12.7)	0.57	0.2, 1.6	0.431
	II	73 (88)	58 (73.4)	3.1	1.4, 7	0.007
	III	3 (3.6)	11(13.9)	0.24	0.07, 0.9	0.047
	IV	1 (1.2)	–	–	–	–
BMI		27.5 (4)	25.9 (3.8)			0.018
Cigarette use		22 (26.8)	16 (20.3)	1.4	0.7, 3	0.358
Pack years		20 (11.6)	28 (20)			0.174
Any alcohol use		50 (61)	27 (32.5)	3.2	1.7, 6.1	<0.001
BPH medications		18 (22)	10 (12.3)	2	0.9, 4.6	0.145
Immunosuppression	Total	5 (6.4)	23 (29.1)	0.17	0.06, 0.47	<0.001
	HIV	4	7			
	Lymphoma	1	5			
	SOTR	–	12			
Duration of immunosuppression	Mean years (SD)	11.6 (8.7)	1.9 (1)			<0.001
Prostate Cancer		9 (10.8)	2 (2.4)	4.9	1, 23.5	0.057
Prostate Cancer Treatment	No treatment	2	–			
	Radical prostatectomy	3	2			
	Brachytherapy	1	–			
	XRT	3	–			
PSA		2.0 (2.2)	2.1 (2.8)			0.902

*: %: percent; BMI: body mass index; BPH: benign prostatic hyperplasia; CI: confidence interval; HIV: human immunodeficiency virus; OR: odds ratio; N: number; PSA: prostate-specific antigen; SD: standard deviation; SOTR: solid organ transplant recipient; XRT: X-ray telescope radiation therapy. ^a^ Non-melanoma controls include 81 cases of squamous cell carcinoma, and 2 cases of basal cell carcinoma.

**Table 2:** Adjusted Results *^,†^.

Variable	*p*-value	OR	95% CI
Age	0.052	1	0.94, 1
BMI	0.011	1.15	1, 1.3
Alcohol use	0.005	3.17	1.4, 7
Skin type II	0.025	1.9	1.1, 3.3
Immunosuppression	0.002	0.127	0.03, 0.5
History of prostate cancer	0.042	11.8	1.1, 129

* BMI: body mass index; CI: confidence interval; OR: odds ratio. ^†^ Variables included in logistic regression model: fixed: age; backward likelihood ratio with threshold of 0.10: BMI, skin type, EtOH use, immunosuppression, history of prostate cancer. Only variables included in final model step are displayed. One binary outcome of Melanoma (yes/no).

### 2.2. Discussion

We assessed the odds of history of prostate cancer among 83 patients with melanoma as compared to time-frame, clinic, and provider-matched 83 controls without melanoma. Our results support the hypothesis that patients with melanoma have higher odds of history of prostate cancer than non-melanoma controls. Additionally, we found that melanoma was significantly associated with body mass index, alcohol use, skin type and immunosuppression.

Both cases and controls fell into the overweight category as their body mass index was higher than 25. However, cases were significantly more overweight. This is consistent with literature showing that melanoma risk is higher in men who are overweight and obese [9].

We also report a significant association between melanoma and alcohol use. Such associations have been inconsistent in literature. Alcohol use may predispose to risky behaviors such as prolonged, unprotected sun exposure, which in itself is a risk factor for melanoma [10]. Rota *et al*. performed a meta-analysis of studies studying the relationship between alcohol use and melanoma [10]. Via a flexible nonlinear meta-regression random effects model which included 16 studies, they found that alcohol consumption was positively associated with risk for cutaneous melanoma with a pooled relative risk for any alcohol use being 1.20 [10]. In univariable analysis melanoma was significantly associated with skin types II and III. This is consistent with current understanding of melanoma pathogenesis as light skinned individuals tend to have higher risk of melanoma than darker skinned individuals due to the density differences in skin melanin [11].

Within our study a significantly high portion of controls were immunosuppressed leading to a significantly lower odds ratio in association to melanoma development. Immunosuppression is a significant risk factor for cutaneous malignancies, especially squamous cell carcinoma [12]. In a prior systematic review of 12 population-based studies reporting melanoma incidence after transplant, overall organ transplant recipients were found to have 2.4 times the risk of melanoma as compared to the general population [12]. Nevertheless, in our study our immunosuppressed patients with melanoma had a significantly longer duration of immunosuppression than the controls suggesting that melanoma may occur at later stages of immunosuppression than non-melanoma skin cancer. Indeed, the mean time for de novo melanoma development ranges from 3–5 years post-transplant [13,14]. Our controls (who were patients with non-melanoma skin cancer) had been assessed very early after the start of their immunosuppression; therefore, they may not yet have had sufficient follow-up time for melanoma development, which lead to the overwhelmingly skewed results and possible Type 1 error.

In univariable and multivariable analyses melanoma was significantly associated with a history of prostate cancer. Androgens have long been implicated in prostatic carcinogenesis and thus, androgen deprivation is the mainstay of prostate cancer treatment [15,16,17,18]. An example of the pathophysiologic relationship is illustrated in severe acne, an inflammatory androgen-induced condition which has been found to be significantly associated with increased risk of prostate cancer [19,20].

The relationship between melanoma and prostate cancer lies in the common androgen-dependence [2,21,22]. Androgens play an important role within the skin, and specifically within melanocytes. Testosterone has been shown to increase melanoma cell proliferation [23]. Moreover, an imbalance of androgens as seen in severe teenage acne may be an integral component in tumorgenesis, and this finding has been supported by Li *et al.* [8].

Secondly, androgens may indirectly induce melanoma development through their effects on the enzyme telomerase. Androgens stimulate telomerase activity, thus promoting elongation of telomeres [24,25]. An increased risk of melanoma has been associated with longer telomeres [26]. Thus, through telomere elongation androgens may expand melanocyte life span and contribute to melanoma development.

Thirdly, androgens may influence melanoma risk through immune system suppression as a blockade of androgen signaling improved survival in melanoma patients [27]. Additionally, as immunotherapy has been shown beneficial in the treatment for prostate cancer and melanoma, a common pathophysiologic mechanism of immune deficiency may be spurring the concurrent development of both cancers [8].

Our study had significant limitations. Our patient sample was derived from a single-provider within an academic institution. This limited us to a small number of cases of melanoma which significantly limited the power of the study. Additionally, since thicker melanomas are sent to surgical oncology by the referring dermatologist for treatment, our cases only included melanomas of up-to 0.60 mm in Breslow thickness which limited our investigation to only superficial melanomas. Our controls were derived from the non-melanoma cases treated by the same provider, within the same clinic and time-frame of treatment as the cases which greatly limited underlying confounding differences between cases and controls. However, the non-melanoma patients within the dermatologic surgery clinic are high-risk patients with increased frequency of immunosuppression which is greater than that observed in the general population. Thus, this limited the generalizability of our results to the general population as we were unable to compare the melanoma cases to absolutely healthy controls.

## 3. Experimental Section

Our retrospective case-control study was performed at the Dermatologic and Mohs Surgery Center of University of California, San Diego. This study was approved by the University of California, San Diego Institutional Review Board.

Power and sample size calculations were performed via the Java Applets for Power and Sample Size for a two proportion Chi Square test. As our data set the proportion (p1) for cases at 0.11, in order to maintain 80% power with a two-sided test, a balanced sample was chosen with an effect size of 10% difference between the two groups (p2 = 0.01) [28]. Alternative sample size calculations were performed in order to detect the optimal control group size, however, there was a relatively small incremental decrease in effect size with substantial increases in control group size—thus, a balanced sample was believed to be the most efficient and informative option.

Cases of melanoma and non-melanoma were assessed via electronic and paper medical records, as well as the clinic billing records via ICD-9 diagnosis codes. Cases were selected if the patients had a diagnosed melanoma within a four year period between 2010 and 2014, were male, and had accessible medical records. The final group of cases represented a group of unique men—none of the subjects was represented twice. Controls were chosen from a cohort of patients treated within a similar time frame (two-year overlapping period of 2010–2012 due to availability of data), by the same clinical provider, within the same clinical facility for conditions other than melanoma, including non-melanoma skin cancer (81 cases (98%) of squamous cell carcinoma, and 2 cases (2%) of basal cell carcinoma). The individual control subjects were chosen via random integer set generator which displayed 83 random integers between 1 and 1056. The final chosen 83 controls did not include any of the same patients who were in the cases group; thus, the two groups (cases and controls) were independent of each other.

Primary outcome was melanoma development defined as having any diagnosis of melanoma *in-situ*, or invasive melanoma. Lifestyle variables such as alcohol and cigarette use were gathered from self-reported data within the medical record where available. Immunocompromised state was defined as having a solid organ transplant (kidney, liver, heart or lung), being on chronic immunosuppressive therapy, chemotherapy, having a diagnosis of blood cancer (leukemia or lymphoma), or being HIV-positive. Cigarette use was defined as any use within 6 months of the surgical procedure for melanoma. Alcohol use was defined as any current, ongoing, self-reported alcohol use within 6 months of the surgical procedure for melanoma. The reported prostate-specific antigen is the value from the most recent laboratory draw to the surgical procedure for melanoma with a maximum time period of 1 year. Benign prostatic hyperplasia medications included were alpha-blockers (terazosin), 5-alpha reductase inhibitors (dutasteride, finasteride), and phosphodiesterase-5 inhibitors (tadalafil, sildalafil). Skin type was assessed via the Fitzpatrick scale which classifies skin by its response to ultraviolet light. Skin type I includes patients with pale white skin who always burn, and never tan. Skin type II includes fair patients who only minimally tan and usually burn. Skin type III is a common skin type which can tan uniformly and only sometimes burn. Skin types IV–VI are darker skin tones that rarely burn and tan well to easily [29]. Melanomas were classified by their size (area), anatomic location, histologic type, Breslow depth and Clark level. Clark level I includes melanoma *in-situ* (confined to epidermis); Clark level II melanomas invade into the papillary dermis; Clark level III includes invasion through the whole papillary dermis; Clark level IV includes invasion past the papillary dermis into the reticular dermis; Clark level V is invasion into subcutaneous fat [30].

The data were analyzed via Independent T-test for continuous variables, Fisher’s exact test for binary variables, and logistic regression model with backward likelihood ratio technique with removal set at *p* = 0.1, in SPSS Version 21 (SPSS, Inc, Chicago, IL, USA). Factors that were significant predictors of melanoma in univariable analysis were included in a multivariate analysis. In all cases, *p* < 0.05 was considered statistically significant. The primary outcome of interest was the development of melanoma, the primary exposure was history of prostate cancer. Confounding covariates included skin type, age, immunosuppression, body mass index, and alcohol use.

## 4. Conclusions

Our single-academic provider case-control study found that melanoma is significantly associated with history of prostate cancer. Although our results do not confer any causal relationships, they suggest that in addition to routine skin-checks, and family history analysis, melanoma screenings may benefit from including questions on prostate cancer history for male patients.

Future large prospective studies are necessary to elucidate on the potentially causative or symbiotic relationship between prostate cancer and melanoma, and what role other risk factors, such as immunosuppression duration, play in the association.
